# Long−Distance Wind Dispersal Drives Population Range Expansion of *Solidago canadensis*

**DOI:** 10.3390/plants11202734

**Published:** 2022-10-16

**Authors:** Zheng Zhang, Guangyue Wen, Dexiao Bu, Guojun Sun, Sheng Qiang

**Affiliations:** 1 Weed Research Laboratory, Nanjing Agricultural University, Nanjing 210095, China; 2 Bureau of Agriculture and Forestry of Jintan District, Changzhou 213200, China

**Keywords:** diaspore dispersal, wind direction, wind speed, dispersal distance, spatial pattern, plant establishment

## Abstract

Canada goldenrod (*Solidago canadensis* L.) is a serious invasive alien plant species that exerts negative effects on natural and agricultural ecosystems in China. Few studies have addressed the dispersal of *S. canadensis* to explain how it rapidly spreads to large areas over long distances. Here, we quantify the dispersal of *S. canadensis* via wind by capturing in situ−stained diaspores. The diaspores were trapped and counted along 11 radiating transects from the center of a dispersal source. *Solidago canadensis* diaspores could be dispersed in all directions from the source, traveling longer distances and in greater amounts in the downwind direction than the upwind one. With a source including about 58 million diaspores and a wind speed at Beaufort scale 4, the dispersal distance in the prevailing wind direction (PWD) was at least 2000 m. Diaspores shattered at a rate of approximately 3% daily with the common wind speed of scale 4, indicating that dispersal could last for more than a month. A mechanistic model was used to fit the dispersal curve along the PWD. Although the model slightly underestimated long−distance dispersal, it still demonstrated the potential of long−distance dispersal with great source strength. Wind−dispersed diaspores to new areas persisted over winter and were able to form new plants at a density of about 2 plants per m^2^ at 500 m away from the source. Further experiments showed that the dispersed amount of *S. canadensis* diaspores was significantly positively correlated with the temperature and wind speed, but significantly negatively correlated with relative humidity, which indicated that, during a day, the maximum dispersal usually occurred in the afternoon when the temperature was the highest and the relative humidity the lowest. In addition, for an already existent population patch, the patch range can expand 2−4 m per year, mainly depending on the seedlings recruited from the rhizomes. These results provide insights into the long−distance dispersal of *S. canadensis* by wind and its effects on the range expansion process.

## 1. Introduction

Invasive plant species are a significant component of global change with far−reaching consequences for the invaded communities [1,2]. Some non−indigenous species can establish and spread rapidly in new environments to penetrate, alter, and replace native plant communities by outcompeting native taxa, thus reducing local biodiversity. Invasive alien plant species pose a major global threat to biodiversity [3], =endangering species, which could even become extinct [4,5].

The Asteraceae are not only highly successful and the largest eudicot family, but are also notorious for including many members of invasive flora [6], because many of its member have evolved highly refined, wind−dispersed seeds. Many successful invaders in Europe are species belong to this family [7]. Their occurrence in plant communities leads to reduced biodiversity, as well as altered community structure and processes [8,9,10]. In China, 20% of the most serious plant invaders (N = 369) are members of the Asteraceae [11], including Canada goldenrod (*Solidago canadensis* L.), which is listed as one of the most important invasive species by the Ministry of Environmental Protection [12].

*Solidago canadensis* is native to North America and most abundant in the Eastern USA and Canada. It is now widely distributed in Europe, Asia, and Australia after both intentional and unintentional introductions [13,14]. It is a widespread rhizomatous perennial herb, which reproduces sexually as an obligatory outcrosser [15,16] and asexually through rhizome propagation. All the introduced populations of *S. canadensis* are polyploid, which confers enhanced thermal tolerance and sexual reproduction under high summer temperatures in China [14]. Hence, they can grow up to 3–4 m tall and feature stalkless, alternate, lanceolate leaves between 5 and 20 cm long. Inflorescences are pyramidal panicles that hold huge numbers of capitula with yellow disc and ray florets. The seed matures and disperses from September to mid–January. Based on the only official record, *S. canadensis* was introduced to Shanghai as an ornamental plant in 1935 [17]. It has escaped from parks and gardens since the 1980s; it has rapidly spread to Southeast China, including Zhejiang, Jiangsu, Jiangxi, Anhui, Hubei, Taiwan, Sichuan, Hunan, Guangdong, Yunnan, Henan, and Liaoning Provinces. It can often be found in abandoned fields and along roadways. It is now considered to be a serious invasive weed in China, both because of its very rapid range expansion and its negative effects on native ecosystems [18].

Although invasive plant species vary greatly in their morphology and life history, there seem to be some biological traits commonly associated with successful invaders. Plants with fast and profuse germination, fast growth, and high reproductive effort are more likely to become invasive [19]. A plant’s capability of vigorous vegetative reproduction (clonal growth) is another trait that increases the probability of an invader’s success [19,20,21], but for colonizing new sites, the production of numerous, widely dispersed seeds is most crucial [22]. *Solidago* *canadensis* can produce a large number of tiny seeds. A single plant can produce up to 20,000 seeds (diaspores) per year [23]. These diaspores bear pappus, facilitating long–distance wind dispersal. There are many simulative studies of windborne seed dispersal and the factors that influence it, but dispersal studies of invasive Asteraceae in natural environments are limited. Those factors include propagule size, achene type, achene length and width, and pappus length [24,25,26] and wind characteristics, including wind speed [27] and direction. In our study, the dispersal dynamics of *S. canadensis* diaspores by wind in nature were observed by tracing diaspores stained with safranine T [28]. Dispersal distributions and populations established in new fields were further studied to better understand the patterns of the rapid range expansion of *S. canadensis*.

## 2. Results

### The Distribution of Dispersed Diaspores in All Sampled Directions

**Figure 1:**
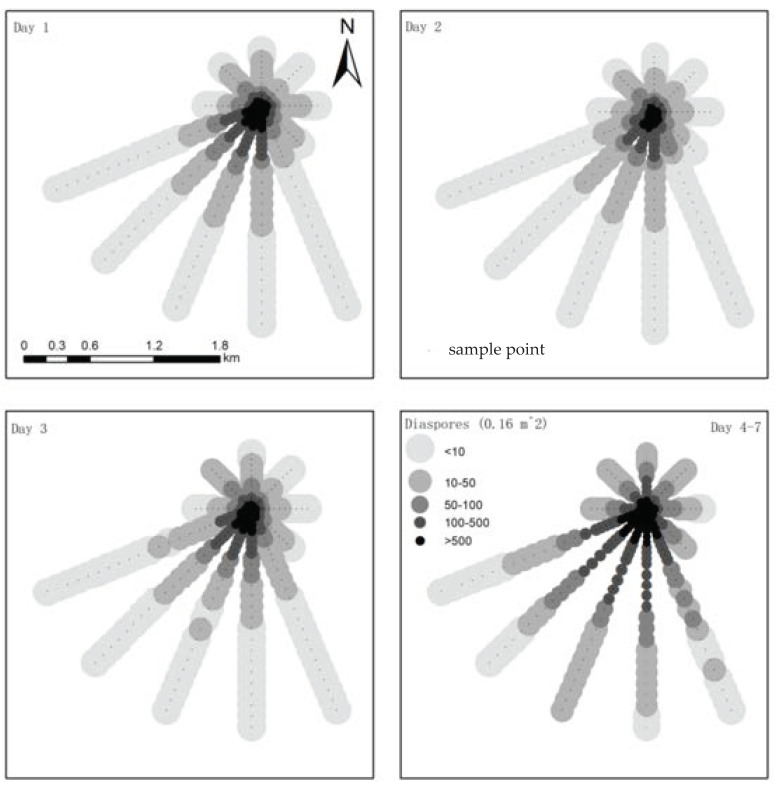
Sampling design of *Solidago canadensis* fields in 2014 and quantity of diaspores collected in each trap on different sampling days. The *S. canadensis* source plants were established at the center of the radiating transects. The prevailing wind direction (PWD) was 22.5° north–northeast. The density of seeds collected at each trap is represented by the gray gradation, and the darker, the higher diaspore density is.

On average, 34,435 ± 9662 diaspores (n = 20) were produced by each *S. canadensis* plant, resulting in a dispersal source of approximately 58 million diaspores. About 68% of the diaspores were collected on traps located <50 m away from the source, 24% between 50 and 100 m, and 8% at distances beyond 100 m (Figure 1). The diaspores were dispersed in all directions from the source, but at different rates. The distance was longer in the PWD than in the upwind direction. At the same distance, many more diaspores were collected in the downwind directions than in the upwind direction.

More diaspores were collected the first and third days than on the second one; diaspores collected on Day 7 were approximately three–times more than on the first day.

**Figure 2:**
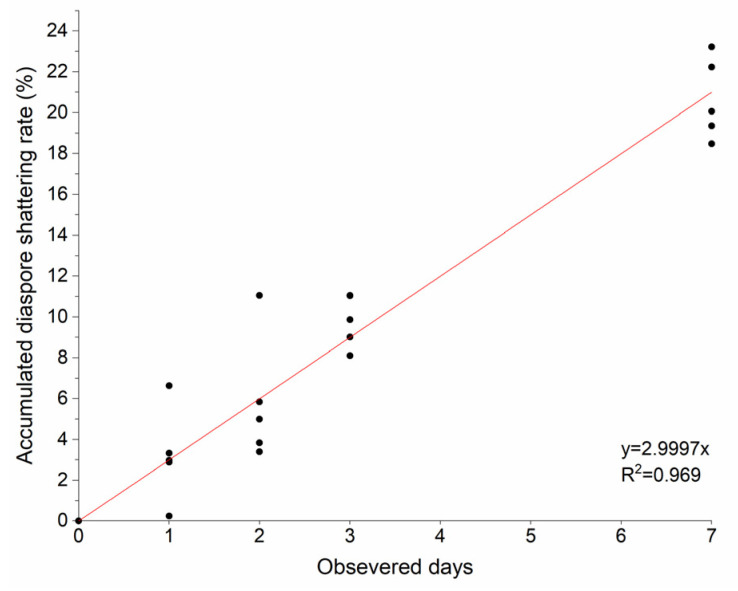
Regression analysis of cumulative diaspore shattering of *Solidago canadensis*.

The cumulative shattering rate increased over time with 20.6% of seeds shattered after seven days. Based on a linear regression model of seed shattering, at a daily rate of 3%, the dispersal period of *S. canadensis* could last for more than one month (Figure 2).

The mean plant height and infructescence length were 2.10 m and 0.31 m, respectively; thus, the mean diaspore release height was 1.79 m, assuming the release point was at the middle of the infructescence. The diaspore strength of 1,887,375 to 6,378,962 was calculated based on the difference in diaspores’ weight before and after a selected dispersal period and the 1000-diaspore weight of 54.10 mg. The average wind speed was 5.68–6.71 m s^−^^1^ during the experiment period, with Beaufort scale 4. (Table 1).

**Figure 3:**
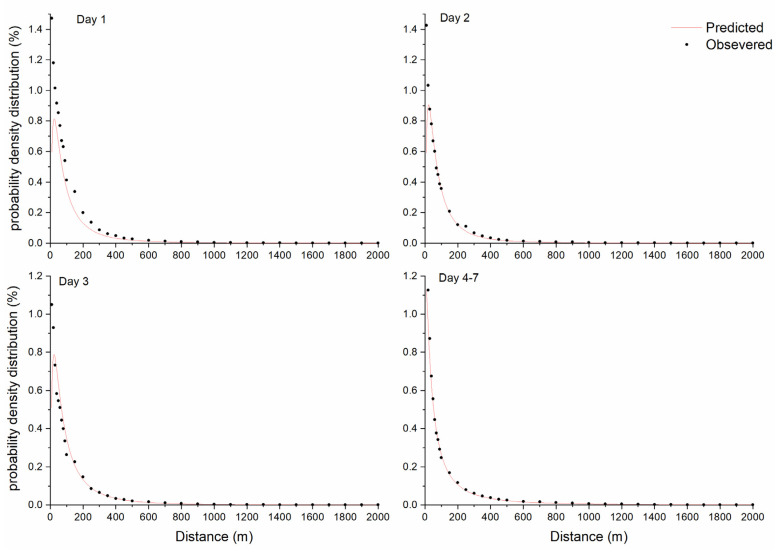
Mechanistic model fitting of probability density for *Solidago canadensis*.

The mechanistic model fit the dispersal curves, but sightly underestimated dispersal density. The dispersal kernel was found near the source, and the probability density decreased sharply within 200 m. Long−−distance dispersal of diaspores (distance ≥1000 m) was observed. In the downwind direction, *S. canadensis* could disperse at a distance of 2000 m with about three diaspores per 0.16 m^2^ for 1 day’s dispersal and 10 diaspores 0.16 m^2^ for 4 days’ dispersal, while the predicted values were 1 and 9 diaspores per 0.16 m^2^, respectively. The predicted amount of long−distance dispersed diaspores was less than the observations. The model predicted that the distribution densities were about two diaspores per m^2^ at a distance of 4000 m and one diaspore per m^2^ at a distance of 5000 m after a 4−day period, suggesting that longer dispersal distances are possible with a strong enough source (Figure 3).

**Figure 4:**
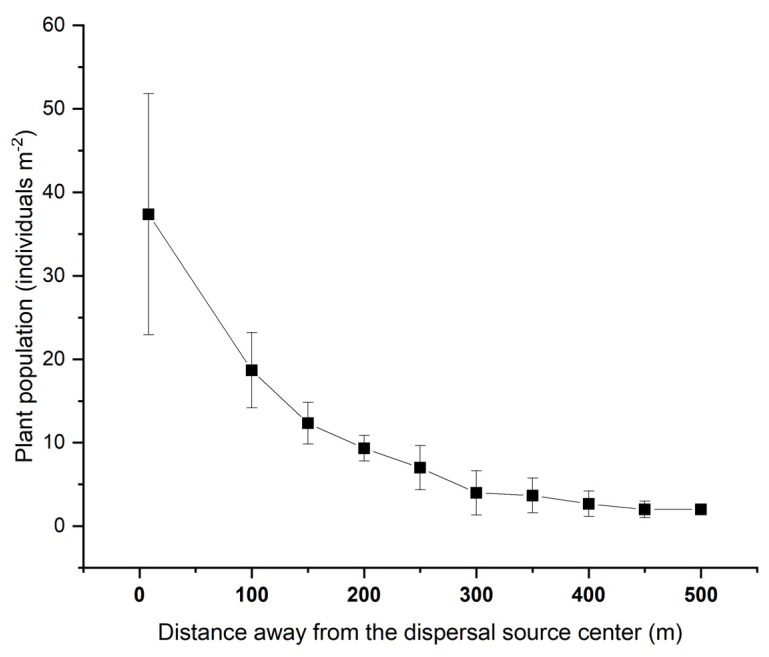
Population densities of *Solidago canadensis* the year following dispersal.

In 2014, beyond the dispersal source, almost no *S. canadensis* plants were found. The next year (2015), *S. canadensis* plants were found around the study plot. Along the dispersal direction, the population densities decreased with increased distances. The plant density was 23 to 65 individuals m^−2^ between 0 and 4 m away from the plot edge (6–10 m away from the plot’s center). At greater distances, there were about 10 and 2 plants per m^2^ found at distances of 200 and 500 m, respectively (Figure 4).

**Figure 5:**
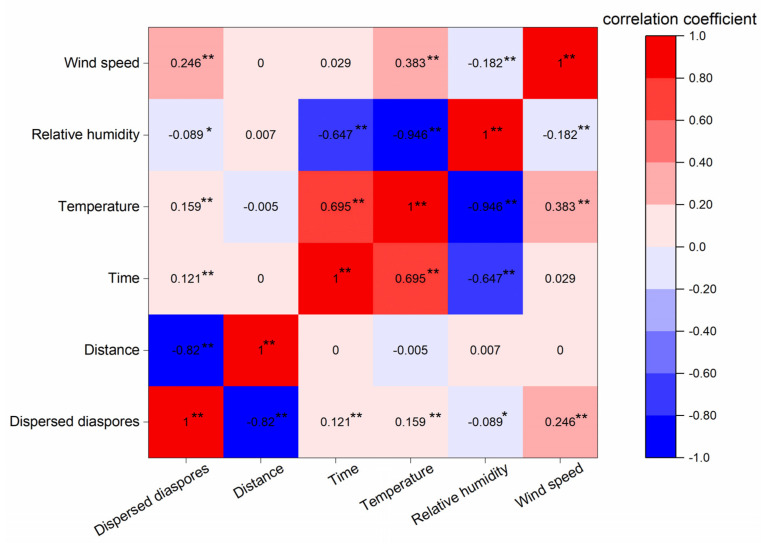
Correlation analysis of relationships between the amounts of dispersed *Solidago canadensis* diaspores and weather factors. *: *p* ≤ 0.05; **: *p* ≤ 0.01.

Our observed results showed that weather condition could influence the *S. canadensis* dispersal process. The dispersed number of *S. canadensis* diaspores was significantly positively correlated with temperature (0.159) and wind speed (0.246) during dispersal, but significantly negatively correlated with relative humidity (−0.089) (Figure 5). The result indicated that, during a day, the maximum dispersal would usually happen in the afternoon when the temperature was the highest and the relative humidity the lowest.

**Figure 6:**
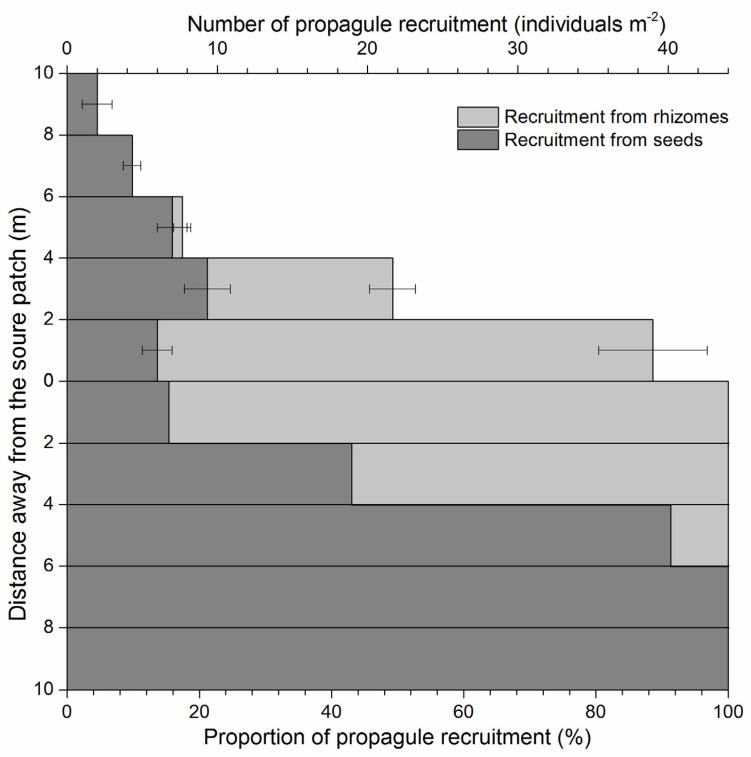
Comparison of *Solidago canadensis* propagules from seeds and rhizomes over distance.

In an already existent patch of *S. canadensis*, new propagules are recruited both from seeds and rhizomes; the closer to the source patch, the more frequently propagules were recruited from the rhizomes. Vegetative propagules comprised 81.97% of new propagules in the area of 0–2 m away from the source patch and 55.77% in the area 2–4 m away, but all new propagules were recruited from seeds in the area >6 m away. The maximum distance from the source patch where vegetative propagules appeared was 4.4 m (Figure 6).

## 3. Discussion

Long–distance dispersal (LDD) is a key mechanism by which many plants spread across landscapes to reach favorable habitats and enter new communities [29,30], which makes it possible for species to invade new sites successfully. Despite the importance of dispersal, there is still a considerable gap between theoretical understanding of spatial processes and empirical studies of real–world systems. This gap is particularly evident for plant communities, where seed dispersal determines most long–distance movement, but is difficult to track directly during LDD, especially to find those seeds that have been long–distance dispersed [31,32,33]. In nature, fruit and seed morphology often indicate the general means of dispersal; diaspores with plumose or comose structures are usually capable of aerial transport [24]. The achenes of *S. canadensis* diaspores with pappi attached are suited for wind dispersal, which is traditionally considered as one of the potential vehicles for the species’ LDD. In this study, the LDD of this species by wind was traced by finding the dispersed diaspores far away from the source with the help of a simple safranine staining method. The light–colored diaspores of *S. canadensis* were marked with striking magenta color after in situ staining, which was obviously different from the unstained ones in color. By using this marking system, the trapped diaspores of *S. canadensis* that dispersed from source plants were easily identified and then quantitated. In our experiment, stained diaspores were found in traps >1000 m away from the source in the downwind direction, providing direct evidence that the LDD of *S. canadensis* via wind is the main dispersal pathway in this region. Additionally, our results indicated that the safranine staining method is a useful tool for easy tracking and retrieving of diaspores for plant–dispersal research.

### 3.1. Seed Dispersal and Seedling Recruitment of S. canadensis

It is generally recognized that seed dispersal represents a critical stage in the life cycle of flowering plants, reducing the adverse effects of high seedling density around the parent plant and promoting colonization of new habitats [34]. Parent plants disperse their offspring to a variety of microenvironments to maximize the chance that some will survive. For *S. canadensis*, it can produce a huge number of wind–disseminated seeds (diaspores), which will disperse away when mature. Wind dispersed *S. canadensis* seeds in all directions around the parent plants into different environments nearby or a long distance away. However, environmental factors influence the distance and direction of seed dispersal, but not all factors within a seed shadow are equally important. Wind had a modest effect on *S. canadensis* diaspore dispersal in that the number of diaspores dispersing outside the source plants gradually decreased with distance, leading to most diaspores being deposited within 50 m of the source. However, most of the total deposited diaspores and increased dispersal distance occurred in the prevailing wind direction. Although the number of *S. canadensis* diaspores dispersing far away from the source was relatively low, a small number of diaspores were directly shown to reach the maximum measured distance of 2000 m on the downwind side of the source patch and 500 m on the upwind side in this study. Distance and direction of seed dispersal affect a seed’s fate [35]. These events taking a small proportion of *S. canadensis* diaspores far away from their parent plants qualify as LDD, which can have impacts at larger (regional) scales, directly affecting spatial spread and colonization rates, giving opportunities for these diaspores to reach remote, but suitable habitats. It was confirmed in this study that, after the diaspores’ arrival at a new appropriate site, e.g., the abandoned field, seedlings would recruit from these deposited diaspores the following year, though with a germination proportion lower than one thousandth of the dispersed diaspores. Once the adult plants establish, the subsequent colonization of the new site can begin. In this study, we observed that, in an already existent patch of *S. canadensis*, the rapid range expansion of the patch depends mainly on the asexual reproduction (propagules recruited from rhizomes) as *S. canadensis* can produce aggressive rhizomes. Patches can expand 2–4 m by plants recruited from rhizomes at a high density, while the farther, scattered plants were all recruited from dispersed seeds. These results provide evidence that the two reproductive patterns of *S. canadensis* (both sexual reproduction by seeds and asexual reproduction by rhizomes) play different roles in the establishment, colonization, and spread of the invasion process. During seedling establishment, polyploidization contributes to the pre–differentiation of the competitive ability among native *S. canadensis* populations, facilitating its invasion in China [14]. Polyploid–promoted allelopathic potential makes these introduced polyploids more competitive against local plants [36].

### 3.2. Factors Influencing Wind Release and Dispersal

It has become clear that, for wind–dispersed plants, diaspore release is the first step in dispersal. Indeed, diaspore release is a crucial determinant of many aspects of subsequent plant movement [37,38]. More specifically, environmental conditions during the final stages of fruit and seed release can significantly affect dispersal distances [38,39], and the probability of reaching favorable sites for germination [40]. We confirmed that the dispersed numbers of *S. canadensis* diaspores were correlated with weather conditions, including wind speed, temperature, and relative humidity. High weed speeds and temperatures promoted diaspore release, while high relative humidity suppressed release. Evidently, highly humid air can physically prevent diaspore abscission, even under wind turbulence conditions, by hindering the opening of the involucres [41] or promoting the closing of drag–producing fibers [42,43]. Further, even when diaspores release from a plant, moist pappi can stick together, reducing the wind–exposed area, and the added weight of moist diaspores significantly increases, resulting in the acceleration of diaspores’ settlement, thus greatly reducing the dispersal distance of diaspores. On the other hand, low relative humidity and high air temperature tend to be correlated diurnally with higher horizontal wind speed and mechanical turbulence, thus favoring LDD [37,44]. To sum up, seed release and LDD in the wild tend to occur in dry and blustery weather with strong turbulence. In Eastern China, where *S. canadensis* has invaded seriously, widespread dry and windy weather conditions during the winter (the maturation and dispersal period of this species) would facilitate the diasporas’ release and wind dispersal, driving the range expansion of invasion.

Based on its reproductive and dispersal characteristics, the following points should be considered to control *S. canadensis* invasion: (1) the sporadic distribution of *S. canadensis* communities, especially in habitats with low disturbance, should be carefully monitored, not only for obvious plants, but also for their early control; the manual pulling of whole plants may be warranted; (2) once the patch of *S. canadensis* has been established, chemical and mechanical control of *S. canadensis* should be undertaken before seed maturation to prevent fructification or remove the inflorescences to stop the wind dispersal of diaspores; (3) for more complete control, rhizomes should also be subject by deep ploughing to prevent new vegetative propagule recruitment.

## 4. Materials and Methods

### 4.1. Study Site

Three experiments on seed dispersal dynamics were conducted, respectively, in different locations in 2014, 2015, and 2016.

Experiment A was carried out in an abandoned field (31.116° N, 121.101° E) located near Huaxu Road in Qingpu, Shanghai, China, in 2014. The selected field was seriously infested with *S. canadensis*. It was surrounded by an open grass area interspersed with a few farmlands. The prevailing wind direction (PWD) during the dispersal window of *S. canadensis* (October to January 2015) was northwest (22.5°).

In late October 2014, prior to diaspore shattering, a study plot (Plot A) with about a 6 m–semidiameter circular covering area was established within the main study area. The *S. canadensis* plants beyond the plot were eliminated. Density determinations of *S. canadensis* in the study plot were made in five 0.5 × 0.5 m quadrats randomly located in the study plot. Additionally, three plants in each quadrat were selected to determine their height, infructescence length, capitulum number, floret number per capitulum, and seed setting rate. Seed production in the study plot was estimated by determining the plant density and individual plant fecundity. 

Experiment B took place in an abandoned field (31.285° N, 120.648° E) situated in Xiangcheng District, Suzhou, Jiangsu Province, China, in 2015. The field was measured to be about 100 × 120 m, and some *S. canadensis* patches were located. One patch measuring about 4 × 4 m located near the field’s center was selected for study (Plot B).

Experiment C was conducted in an abandoned field (31.722° N, 119.437° E) situated in Jintan District, Changzhou, Jiangsu Province, China, in 2016. The 50 × 250 m field was measured, where *S. canadensis* was newly infested with scattered plants. A patch covering about 1.2 × 1.8 m was selected for study prior to seed dispersal. The boundary of the plot was circled by a line. All *S. canadensis* plants beyond the study plot were removed.

### 4.2. Staining In Situ

To facilitate their tracking, all *S. canadensis* diaspores in Experiments A and B were stained in situ in the afternoon of the day before the first sampling (the staining day was designated as Day 0) with a 1% *w*/*v* solution of safranine T (Sinopharm Chemical Reagent Co., Ltd., Shanghai, China) in 50% ethanol using a sprayer. Additionally, five randomly selected plants in Plot A were brought to the lab to determine the total and 1000–seed weight.

In Experiment C, the diaspores of *S. canadensis* plants were stained immediately after the plot was set up.

### 4.3. Seed Sampling

Experiment A: After diaspore staining, seed traps were placed at a range of distances along radiating line transects in eleven directions from the plot center at angles relative to the PWD (in the clockwise direction) at 22.5° intervals. To facilitate sampling, traps located farthest from the source were set at a 500 m distance only in the 167.5°, 112.5°, 157.5°, 202.5°, 247.5°, and 292.5° directions and up to 2000 m away in the other five directions. Otherwise, traps were positioned at 10 m intervals along each transect from the dispersal source to 100 m, at 50 m intervals from 100 m to 500 m, and at 100 m intervals from 500 m to 2000 m. Traps consisted of a square (0.16-m^2^) sticky board coated with tung oil for diaspore adherence, fixed to the ground by nails at four corners. Each trap was assigned a unique code for proper identification. The first sampling was conducted at 16:00 by taking individual digital photographs of each trap before replacing the sticky board with a new one for the next sampling. The same procedure was used for three consecutive days and after seven days at about the same time of the day. Stained seeds recorded in each photograph were counted in the laboratory.

Wind information (wind speed and direction) and temperature were recorded at 1 h intervals from 08:00 to 16:00 with a portable anemometer and a temperature recorder, respectively. Wind data were not gathered at night because organisms typically maximize dispersal by utilizing daytime updrafts [45].

Experiment B: Five concentric arcs covering 90° (main wind direction ± 45°) from 10 to 50 m away from the plot center at 10 m intervals were utilized as the transects for diaspore trapping. Diaspore traps were placed at 10 m intervals along the arcuate transects. In total, 50 traps were set along all five transects. The traps were made of the same materials as used in Experiment A, but with an area of 0.1 m^2^. The study was conducted for three continuous days, with *S. canadensis* diaspores sampled every two hours from 10:00 to 18:00 at 5 time points (10:00, 12:00, 14:00, 16:00, and 18:00). Every morning at 08:00, before the first sampling, the traps were placed at the pre–set positions. Subsequently, the sampled traps were replaced by new ones at every time point. The sampled traps were covered with plastic film and then brought to the laboratory. All *S. canadensis* seeds on the traps were counted. Relative humidity, temperature, and wind speed were measured at half–hour intervals during the sampling period from 08:00 to 18:00.

Experiment C: After diaspore staining, around the patch boundary, five 2 m–wide belt transects were set adjacently at distances of 0–2 m, 2–4 m, 4–6 m, 6–8 m, and 8–10 m separately, away from the patch boundary. In each belt transect, five diaspore traps with a quadrat area of 0.1 m^2^ were randomly placed in each belt transect. Traps were replaced every 7–14 days as weather permitted, from late November (onset of seed release) to early January (approximate cessation of seed release). After removal from the field, traps were covered with plastic film and all *S. canadensis* diaspores were counted.

### 4.4. Plant Investigation of S. canadensis

Population densities at the Experiment A and C sites were assessed in the year following the dispersal studies.

Experiment A: The following year (13 June 2015), the *S. canadensis* population density was measured by using 1 × 1 m quadrats placed at 50 m intervals along three radiating line transects at angles relative to the PWD of 0, 22.5°, and 337.5° at 100–500 m away from the center. Additionally, seedling densities around the dispersal source (at 8 m and 10 m away from the dispersal source center) were measured by using 1 × 1 m quadrats placed in 11 directions (the directions were the same as in Section 4.3 above).

Experiment C: The population densities of *S. canadensis* were investigated the following year on 25 June 2017 using the same belt transects as for diaspore sampling. In each belt transect, three 1 × 1 m quadrats were randomly sampled. Propagules recruited from seeds and rhizomes were counted separately. In addition, the vegetative propagule that was the farthest away from the circled patch was observed, and its distance to the patch was measured.

### 4.5. Data Analysis

The distribution of *S. canadensis* in all sampling point locations were depicted by gray−gradient mapping with ArcGIS 10.5 (ESRI Inc., Redlands, CA, USA).

Diaspore data collected downwind of the source (±22.5° from the PWD) were used for the analysis of distribution curve of *S. canadensis* dispersal. A mechanistic model [46] as shown below was used, based on the following equation (Equation [1]):

(1) $$\frac{dQ}{dx}=\frac{Q}{x\sigma_{u}\sqrt{2\pi }}exp\left\{-{\left[\frac{ln\left(\frac{xF}{Hu_{H}}\right)}{\sigma_{u}\sqrt{2}}\right]}^{2}\right\}$$

where *dQ/dx* is the frequency distribution of diaspores at distance *x*; *Q* is the source strength (diaspores); *σ_u_* is the standard deviation of the mean wind speed (m s^−1^) at mean seed release height; *H* is the mean seed release height (m), which was estimated by plant height and infructescence length; *F* is the settlement velocity (m s^−1^) of *S. canadensis*, which was obtained from [47].

Linear regression analysis of the shattering rate for *S. canadensis* was performed by using OriginPro 9.0 (OriginLab Corporation, Northampton, MA, USA).

Correlation analysis was used to detect the relationships between the numbers of dispersed diaspores and environmental factors (temperature, relative humidity, and wind speed) by using SPSS 26.0 (IBM SPSS Statistics, New York, NY, USA).

## Figures and Tables

**Table 1:** Values of parameters for modeling of *Solidago canadensis* dispersal.

Parameters (Unit)	Day 1	Day 2	Day 3	Day 7
*Q* (seeds)	1,887,375	1,535,267	2,346,756	6,378,962
*H* (m)	1.79	1.79	1.79	1.79
*σ_u_* (m s^−1^)	1.11	1.09	1.14	1.48
*U_H_* (m s^−1^)	6.46	5.78	6.71	5.68
*F* (m s^−1^)	0.141	0.141	0.141	0.141

## Data Availability

The data presented in this study are available upon request from the corresponding author.
